# Study regarding the survival of patients suffering a traumatic cardiac arrest

**Published:** 2015

**Authors:** V Georgescu, O Tudorache, M Nicolau, V Strambu

**Affiliations:** * Department of Anaesthesia and Intensive Care, “Carol Davila” Nephrology Hospital, Bucharest, Romania; ** Department of Anaesthesia and Intensive Care, “Agrippa Ionescu” Emergency Hospital, Bucharest, Romania; *** Department of Surgery, “Carol Davila” University of Medicine and Pharmacy, Bucharest, Romania

## Abstract

Severe trauma is the most frequent cause of death in young people, in civilized countries with major social and vital costs. The speed of diagnostic decision making and the precocity of treatment approaches are both essential and depend on the specialists’ colaboration.

The present study aims to emphasize the actual situation of medical interventions in case of cardiorespiratory arrest due to trauma. 1387 patients who suffered a cardio respiratory arrest both traumatic and non-traumatic were included in order to point out the place of traumatic arrest.

Resuscitation of such patients is considered useless and resource consumer by many trauma practitioners who are reporting survival rates of 0%-3.5%. As the determinant of lesions, trauma etiology was as it follows car accidents – 43%, high falls – 30%, suicidal attempts – 3%, domestic violence – 3%, other causes – 21%.

Hypovolemia remains the major cause of cardiac arrest and death and that is why the efforts of emergency providers (trauma team) must be oriented towards “hidden death” in order to avoid it. This condition could be revealed and solved easier with minimal diagnostic and therapeutic maneuvers in the emergency department.

## Background

Severe trauma has become the most frequent cause of death in young people, in civilized countries. For this reason, it is obvious that a proper approach in the Emergency Department becomes mandatory and with a maximum impact upon the patient’s survival.

The speed of diagnostic decisions and the precocity of treatment approaches are both essential and depend on the specialists’ colaboration and the existence of an algorithm in which everyone has the chance to apply his/ her experience for the patient’s maximum benefit.

The present study aims to emphasize the actual situation of medical interventions in case of cardiorespiratory arrest due to trauma and the importance of prompt and well-oriented maneuvers that can prevent cardiac arrest installation. 

Resuscitation of such patients is considered useless and resource consuming by many trauma practitioners who are reporting survival rates of 0%-3.5% [**1**-**3**,**7**]. Everybody in the world knows the importance of Advanced Trauma Life Support guidelines of the American College of Surgeons in establishing the order of operations when treating multiple trauma patients, but their recommendations for the interruption of resuscitation or not to resuscitate cannot be applied everywhere and in every situation [**4**].

That is why, in this study, except for patients who were already dead at the moment of their arrival in the hospital, we considered that everybody had at least one chance to be resuscitated [**11**].

## Material and Method

The study included the patients who suffered both traumatic and non-traumatic cardio respiratory arrest in order to point out the place of traumatic arrest among the total cardiac arrests. 1387 patients were admitted between January 2007 and December 2012. The individual parameters followed were: age, gender, place of cardiac arrest, causes of cardiac arrest, and were processed by using Excel 2007. Trauma patients were considered those who suffered car accidents, violence, burns, electrocution, hanging, downing.

The patients were admitted in the Emergency Department of “Sf. Pantelimon” Emergency Hospital in Bucharest and received medical assistance from a complex team made up of emergency physicians, surgeons, orthopedists and neurosurgeons, critical care specialists, cardiologists, imagistic specialists trained in emergency care with at least 5 years of experience.

## Results

The distribution of patients according to each year of study was the following: **2007** – 192, **2008** – 198, **2009** – 231, **2010** – 240, **2011** – 267, **2012** – 259. 

**Fig. 1:**
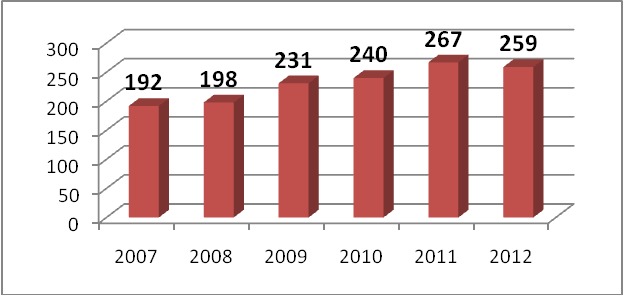
Cardiac arrests distribution according to years

Gender distribution was suggestive with male predominance – 924 and 463 women

**Fig. 2:**
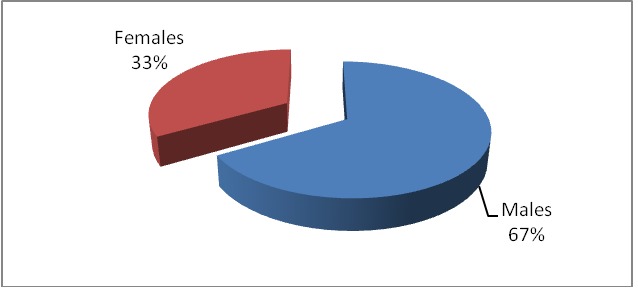
Distribution according to gender

Regarding the distribution on age groups, most patients were part of the 61-80 group, followed by those who were 41-60, and the average age was 54+/- 1,3, slightly raised for women.

**Table:** 

Age group	Patients
1-20 years old	106
21-40 years old	242
41-60 years old	380
61-80 years old	455
over 80 years old	204

**Fig. 3:**
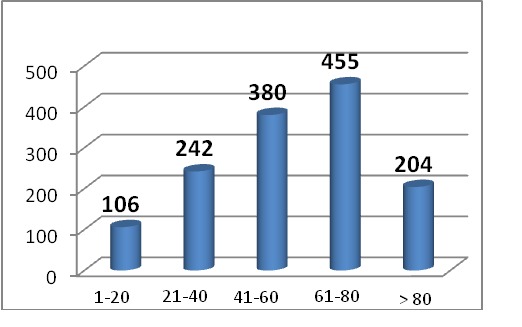
Age groups distribution

Most patients – 862 out of 1387 were living in an urban environment, which could be explained by an increased accesibility to medical servicies and to emergency assistance, but the differences of mentality could also be involved. In both urban and rural environments, males predominated over women – 619/ 289 in urban and 305/ 174 in rural area. This can be explained by a more active and dangerous life experienced by men and a more agresive behavior from their part.

**Fig. 4:**
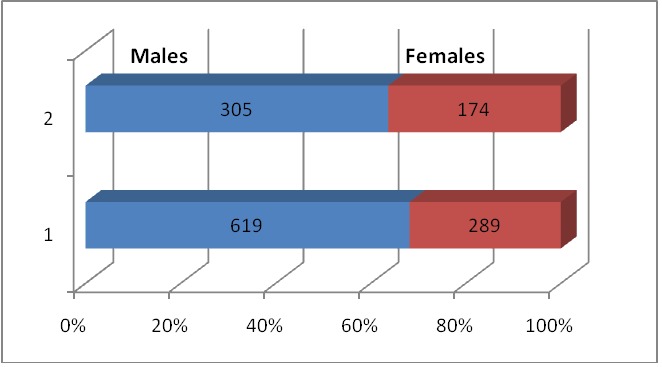
Comparative gender distribution in urban and rural areas

Considering the predominant place the cardiac arrest was encountered, this appeared to be the street, followed by the place of living, public spaces, medical institutions, place of work, in this order. These results correlated with the most frequent age at which cardiac arrest took place, namely the persons most active both from a social and a professional point of view.

**Fig. 5:**
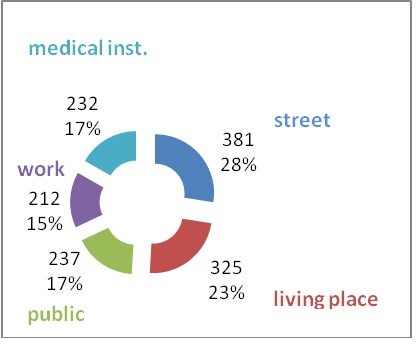
Place of cardiorespiratory arrest

**Fig. 6:**
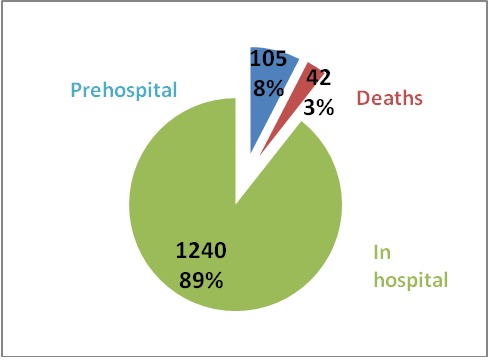
Resuscitation place

Out of 1387 patients, 105 were resuscitated in the prehospital field, 42 died during transport and 1240 were resuscitated in the emergency department.

Regarding the first rithm recorded before resuscitation, in the out of hospital field, this was asistole for most of the cases, followed by ventricular fibrilation, pulseless electrical activity and then ventricular tachicardia. For those who were resuscitated in the Emergency Department, most presented with ventricular fibrilation as the first monitored rithm, follwed by asistole, pulseless electrical activity and ventricular tachicardia [**21**].

**Fig. 7:**
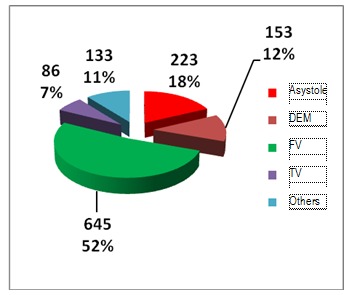
Cardiac arrest rithm of in hospital resuscitated patients

**Fig. 8:**
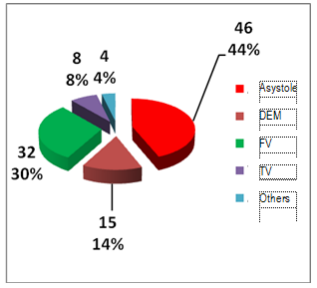
Cardiac arrest rithm of out of hospital resuscitated patients

It could be easily observed that the percentage of cases of asistole was higher in case of out of hospital resuscitation patients when compared with those resuscitated in hospital areas and this was probably because the coronary disease was the main cause of cardiac arrest, as compared with more diverse pathology and causes which characterized the second situation.

There were certain patients who suffered at least one more cardiac arrest. In the group represented by prehospital cardiac arrest, the dominant rithm was asistole, as it can be seen below. This characterizes even the hospital group but the numbers are different.

Ventricular tachicardia was present in a slightly high percentage than the first rithm monitored at the first cardiac arrest, sustaining the idea that cardiac arrest could occur through pulseless ventricular tachicardia which can rapidly turn into ventricular fibrilation.

**Fig. 9:**
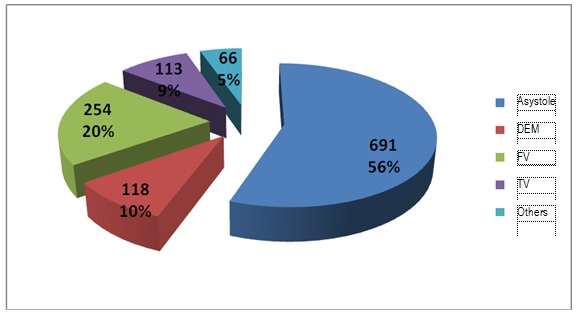
Rithm before the 2nd cardiac arrest – in hospital

**Fig. 10:**
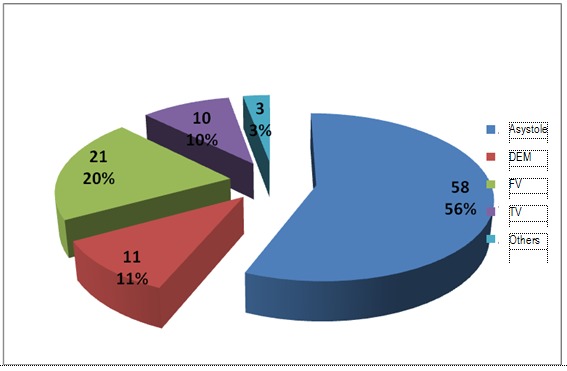
Rithm before the 2nd cardiac arrest – out of hospital

Regarding the ethiology of cardiac arrest in our group, most of cases presented with cardiovasccular disease, followed by respiratory diseases, trauma, cerebrovascular diseases, asphixia, toxic ethiology and suicidal attempts. Considering the last three as traumatic events because they were encountered in such conditions, most of them will go up on the second place in this clasification due to traumatic causes.

**Fig. 11:**
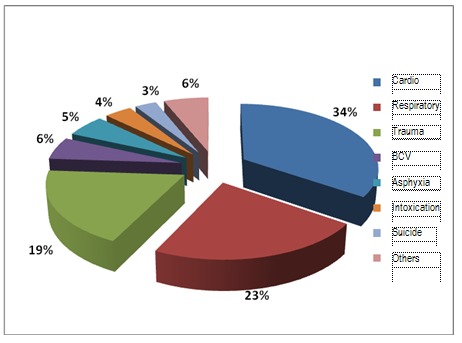
Causes of the cardiac arrest in the studied group

From 261 cases of trauma, 89 presented with cardiovascular lesions.

**Fig. 12:**
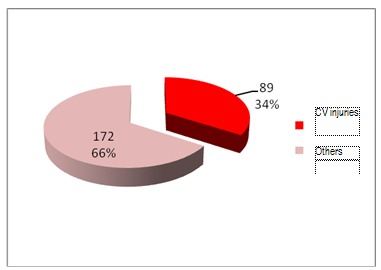
Cardiovascular injuries of total trauma

**Injury Severity Score (ISS)**

Injury Severity Score, introduced by Baker in 1974, is an anatomic score which calculates a total score for multiple injured patients [**12**,**13**,**17**,**18**]. A certain value is atributed to each lesion of a anatomic region. Human body is made up of six regions: Head and Neck, Face, Chest, Abdomen, Extremities (including pelvis), Skin [**17**,**18**]. The highest score is used in each region. The first three scores in the three regions are multiplied by themselves and summed to obtain the ISS. The ISS value can vary between 0 and 75. If there is a region with a score of 6, then the ISS becomes 75 automaticaly. ISS corelates liniary with mortality, morbidity, hospital period [**17**,**18**].

 Although ISS corelates with mortality, it has nothing to do with the amount of resources used in trauma management, such as fluid resuscitation, invasive monitoring of the central nervous system, emergency interventions and cannot be used as a sole criteria to defy major trauma [**12**-**14**,**19**,**20**]. 

**New Injury Severity Score (NISS)**

Was proposed in 1997 and it emerged from the summing of the highest three squared values of AIS without taking into account the anatomic region [**22**,**23**]. NISS can be calculated much easier and can predict surviving with a high accuracy.

**Fig. 13:**
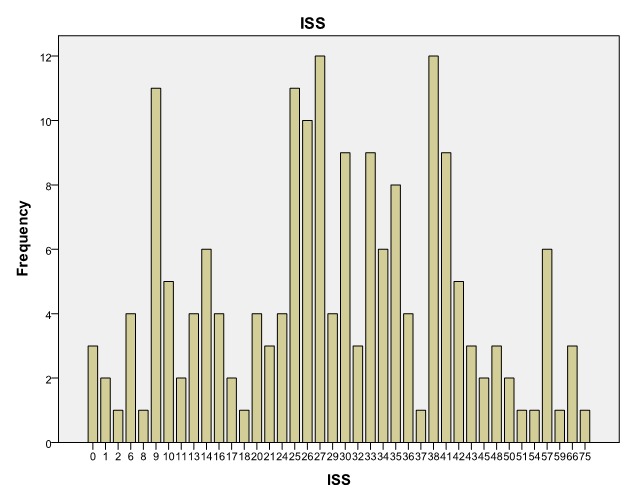
Injury Severity Score (ISS)

In our study, there was no significant statistical corelation between ISS value and the presence of cardiac arrest [**9**], the average value for ISS being 29, 27±13, 95.

Regarding survival, 126 patients without cardiac arrest have survived and 9 out of 261 with cardiac arrest, as well, which means 3, 44%, which corelates with other studies [**4**]. 48 out of these patients have survived the first cardiac arrest event and the 9 who were discharged come from that group. The main cause of cardiac arrest was hypovolemia in the 4 cases of those who survived, hypoxia and severe cerebral trauma in 2 cases, and pneumothorax in one case.

## Discussions and Conclusions

This study was an observational and retrospective one. Even if there were no data available regarding the long-term survival on discharge, it offered some results that could be interpreted as suggestive for rates of survival in cases of a certain lesion [**5**]. In the same time, it created an image on what happens with a traumatized patient in the Emergency Services. According to a recent study, the survival of trauma patients after resuscitation is still uncertain, with poor results that vary between 0 and 3, 47% [**4**].

Most frequent causes of trauma in our group of patients were hypovolemia, severe cerebral trauma, hypoxia, tension pneumothorax. 

As the determinant of lesions, trauma etiology was the following: car accidents – 43%, high falls – 30%, suicidal attempts – 3%, domestic violence – 3%, other causes – 21% [**15**,**16**]. Regarding lesions type, the following could be met: cerebral trauma of different grades – 57%, thoracic lesions – 30%, extremities – 37%, abdomen and pelvis – 17%, spinal lesions – 14% [**15**,**16**].

The unsuccessful resuscitation of patients with traumatic cardiac arrests similar with other recent data emerged from different studies [**6**,**7**,**10**]. No matter the cause, the success in this situation is not as good as the cardiac arrest in out of hospital field.

Regarding the neurologic outcome at the discharge moment, even in our group, the best results were registered by those who suffered cardiac arrest induced by hypoxemia [**1**], followed by those with penetrating thoracic trauma who necessitated minimal interventions in the emergency department, without big thoracotomy, which was performed in the operating room and which announced greater traumatic and vital lesions [**1**,**4**,**8**]. 

This study was somehow limited and its results should be interpreted by taking into consideration the aspect that it was developed in one single center and the number of patients was relatively small. Nevertheless, the results were comparable with the ones in other similar studies, which used even smaller groups of patients and could represent a landmark for future studies. Essentially, the applicability of a certain algorithm should be adapted to local conditions and should include the close collaboration between all specialists involved. The algorithm must have, as a central element, resuscitation, and preservation of vital signs. 

As a conclusion, this study, which was similar to others when outcomes were analyzed, confirms the poor survival rate of the trauma – cardiac arrest combination. Hypovolemia remains the major cause of cardiac arrest and death and that is why the efforts of emergency providers (trauma team) must be oriented towards the early diagnosis of potentially lethally lesions before cardiac arrest. Survivors are selected, with different chances, mostly from the subgroups of trauma patients potentially dead and a special one is that represented by hypoxia. This condition could be easily revealed and solved with minimal diagnostic and therapeutic maneuvers in the emergency department and even on scene: asphyxia, foreign bodies in the upper airway, tension pneumothorax.

**Acknowledgement**

This paper is supported by the Sectorial Operational Programme Human Resources Development (SOP HRD), financed from the European Social Fund and by the Romanian Government under the contract number POSDRU/159/1.5/S/132395. 
